# Permutation Entropy Based on Non-Uniform Embedding

**DOI:** 10.3390/e20080612

**Published:** 2018-08-17

**Authors:** Mei Tao, Kristina Poskuviene, Nizar Faisal Alkayem, Maosen Cao, Minvydas Ragulskis

**Affiliations:** 1 Department of Engineering Mechanics, Hohai University, Nanjing 210098, China; 2 Center for Nonlinear Systems, Kaunas University of Technology, Studentu 50-146, LT-51368 Kaunas, Lithuania

**Keywords:** permutation entropy, ordinal pattern, attractor embedding, multi-dimensional phase space

## Abstract

A novel visualization scheme for permutation entropy is presented in this paper. The proposed scheme is based on non-uniform attractor embedding of the investigated time series. A single digital image of permutation entropy is produced by averaging all possible plain projections of the permutation entropy measure in the multi-dimensional delay coordinate space. Computational experiments with artificially-generated and real-world time series are used to demonstrate the advantages of the proposed visualization scheme.

## 1. Introduction

Real-world time series and experimental data are usually contaminated with noise. System states of such time series are usually complex, nonstationary and difficult to identify. Computation of the complexity for a given time series helps to quantify the intricacy of the model that governs the evolution of that series.

Different well-known complexity measures can be used to describe a time series, including Lempel-Ziv complexity [1] and Kolmogorov complexity [2]. Chaotic features of a time series can be quantified by assessing its maximal Lyapunov exponent [3]. The complexity of a time series can be assessed by dimension estimation models (such as the information dimension [4] and the fractal dimension [5]); also by entropy assessment methods (such as Shannon entropy [6], approximate entropy [7], K-S entropy [8,9], sample entropy [10], conditional entropy [11], multiscale entropy [12]).

Arguably, the most powerful tool to measure the complexity of a time series is the permutation entropy (PE). Since its introduction in 2002 by Bandt and Pompe in their foundational paper [13], it has been successfully applied in a wide range of scientific areas and for a vast number of purposes [14]. The main advantages of PE are: (i) it is simple to use; (ii) calculations are fast; and (iii) it is robust against noise. PE is based on the permutation patterns or the order relations among values of a signal [15]. PE compares the order of neighboring relative values, rather than apportioning amplitudes according to different levels [15,16]. PE can be computed using fast PE algorithms [17]. These characteristics make PE an appealing tool used in a large number of real-world signal and image processing applications [18].

PE is based on one-dimensional time series reconstruction into a *D*-dimensional space with the embedding delay τ. The time series embedding scheme into the higher dimension space was first presented by Packard, Crutchfield, Farmer and Shaw [19,20]. This embedding scheme represents the optimal properties of a dynamical system if the embedding dimension Dand time delay τ (the difference between consecutive observations) are estimated adequately. Theoretical foundations by Takens [21], and expansions of his ideas by Sauer et al. [22] indicate that the embedding dimension of D>2m+1 (where *m* is the fractal dimension of the attractor) almost always ensures the reconstruction of the topology of the original attractor [20]. This surprising result states that time series output is sufficient to obtain complete information about hidden states of the dynamical system [23].

Commonly-used methods for finding the optimal time delay τ are the average mutual information method [24], the correlation sum method [25], the phase space expansion method [26,27] and the geometry-based method [28].

The selection of the optimal embedding dimension is usually based on the examination of some invariant on the reconstructed attractor. Usually, these invariants represent the dynamics of the system and are based on the geometrical properties of that system. An invariant value is computed by increasing the embedding dimension until that invariant value settles down [29]. Typical examples of such methods are the box-counting method [20,30], the correlation dimension method [25,31], the largest Lyapunov exponent method [32], the Kolmogorov–Sinai entropy method [33] and the false nearest neighbors (FNN) method [34,35].

The identification of the optimal embedding dimension *D* and the optimal time delay τ helps to reconstruct the attractor in the delay-coordinate space. However, it is well known that non-uniform embedding (when time delays are not equal) might lead to a better reconstruction of the attractor if compared to uniform embedding (when all time delays are equal) [36,37]. However, the selection of the optimal vector of time delays for non-uniform embedding is a difficult optimization problem that requires massive computational resources. Several approaches have been proposed to tackle this problem. The identification of a near-optimal vector of time delays employing genetic algorithms was proposed in [38,39]. Good near-optimal non-uniform embedding results were obtained by using the ant colony optimization algorithms reported in [40]. Non-uniform time series embedding with special target functions based on the Fourier spectral analysis were presented in [41,42].

The main objective of this paper is to employ non-uniform time series embedding for the construction of a visualization scheme for PE. The optimal embedding dimension and the set of optimal time lags are used to design a computational algorithm for plotting PE as a single surface. The paper is structured as follows. The normalized PE and the target function used to identify optimal time delays are introduced in Section 2. The proposed visualization scheme for PE is introduced in Section 3. The results of computational experiments with the sine wave, the Rossler time series and a real-world time series are discussed in Section 4. A discussion and concluding remarks are given in the last section.

## 2. Preliminaries

### 2.1. Permutation Entropy

For a given time series $\left\{x_{1},x_{2},\ldots ,x_{N}\right\}$, uniform embedding yields a trajectory matrix:

(1) $$Y_{t}=\left\{x_{t},x_{t+\tau },x_{t+2\tau },\ldots ,x_{t+(D-1)\tau }\right\},\;\;t=1,2,\ldots ,N-\left(D-1\right)\tau ,$$

where *D* is the embedding dimension and τ is the time delay. PE quantifies the statistics of ordinal permutations in the rows of the trajectory matrix [29].

For example, the sequence {3,7,6} has ordinal pattern π=1;3;2, since its $x_{1}\leq x_{3}\leq x_{2}$. The ordinal pattern of the sequence {6,3,7} is π=2;1;3. As a consequence, there are D! possible pattern orders, which represent all unique orderings (permutations $\pi_{i},i=1,2,\ldots ,D!$). The relative frequency of each distribution with which they occur in the trajectory matrix is defined as follows:

(2) $$P_{i}=\frac{f(\pi_{i})}{N-(D-1)\tau },\;\;i=1,2,\ldots ,D!,$$

where $f(\pi_{i})$ represents the occurrence number of the $\pi_{i}$ pattern order. Normalized PE is defined here as:

(3) $$H_{D}\left(\tau \right)=-\frac{1}{\ln(D!)}\sum_{i=1}^{D!}P_{i}\ln\left(P_{i}\right).$$

The range of PE values defined by Equation (3) is from 0–1. PE depends on two predefined parameters: the embedding dimension *D* and the time lag τ.

### 2.2. Non-Uniform Embedding

The trajectory matrix produced by non-uniform embedding reads:

(4) $$Y_{t}=\left\{x_{t},x_{t+\tau_{1}},x_{t+\tau_{1}+\tau_{2}},\ldots ,x_{t+\Delta }\right\},\;\;t=1,2,\ldots ,n-\Delta ,$$

where *n* is the length of the observable time series; Δ is the width of the observation window; $\Delta =\tau_{1}+\ldots +\tau_{D-1}$; $\left\{\tau_{1},\tau_{2},\ldots ,\tau_{D-1}\right\}$ is the vector of time delays.

The optimal time delays are computed as the (D−1)-dimensional argument of the following maximization problem [43]:

(5) $$\max_{1\leq \tau_{1},\ldots ,\tau_{D-1}\leq L}\left(\frac{1}{\left(n-\Delta \right)\sqrt{D}}\sum_{k=1}^{n-\Delta }\sqrt{x_{k}^{2}+x_{k+\tau_{1}}^{2}+\ldots +x_{k+\Delta }^{2}}\right).$$

where *L* is the upper range for all time lags.

## 3. The Proposed Visualization Scheme for PE

Visualization of PE as a function of two time delays (at D=3) is proposed in [44]. Such an approach enables one to plot PE as a surface, and the graphical features of that surface leak the underlying complexity of the analyzed time series. Visualizing higher-dimensional arrays is a considerable challenge, and a scheme for rationalizing the information contained in these arrays is of considerable practical benefit, since in practice, one often wishes to use D>3. The proposed visualization scheme for PE at D>3 reads:

1. Determine the optimal embedding dimension *D* for a given time series.
2. Set *L* (the upper range for all time lags). Determine the set of optimal time lags $\tau_{1}^{★},\tau_{2}^{★},\ldots ,\tau_{D-1}^{★}$ according to Equation (5).
3. Repeat in lexicographical order and construct planar images of PE:
  (a) Freeze D−3 optimal time lags;
  (b) Vary two time lags from 1–*L*, and compute PE according to Equations (2) and (3);
  (c) Construct a two-dimensional digital image of PE.
4. Average all (D−1)(D−2)/2 planar digital images of PE.

**Example** **1.** *In the case of D=5, the visualization procedure can be illustrated by a schematic diagram in Table 1.*

Note that only two different time delays $\left(\tau_{k},\tau_{l}\right)$ are not fixed in each plane projection of $H_{D}$. In other words, $H_{D}$ is a two-dimensional image of PE when all time delays $\tau_{s}$, s≠k, s≠l, k≠l are fixed to the corresponding components of the vector of optimal time delays. Finally, a two-dimensional averaged digital image of PE is computed as an arithmetic average of all planar PE images. The upper range *L* should be high enough to ensure that the maximum point of the target function (Equation (5)) does not lie on the boundary of the search space (all optimal time lags should be lower than *L*).

Note that the computation of all possible digital images of PE is not necessary. It is possible to compute the averaged two-dimensional digital image (a square matrix of L×L pixels) of PE directly:

(6) $${\bar{H}}_{D}\left(\zeta =i,\eta =j\right)=\frac{2}{\left(D-1)(D-2\right)}\sum_{k=1}^{D-2}\sum_{l=k+1}^{D-1}H_{D}\left(\tau_{k}=i,\tau_{l}=j\right),$$

where ζ and η are coordinates of a pixel in the *x*- and the *y*-axis; i,j=1,…,L.

## 4. Computational Experiments

### 4.1. The Sine Wave

**Figure 1:**
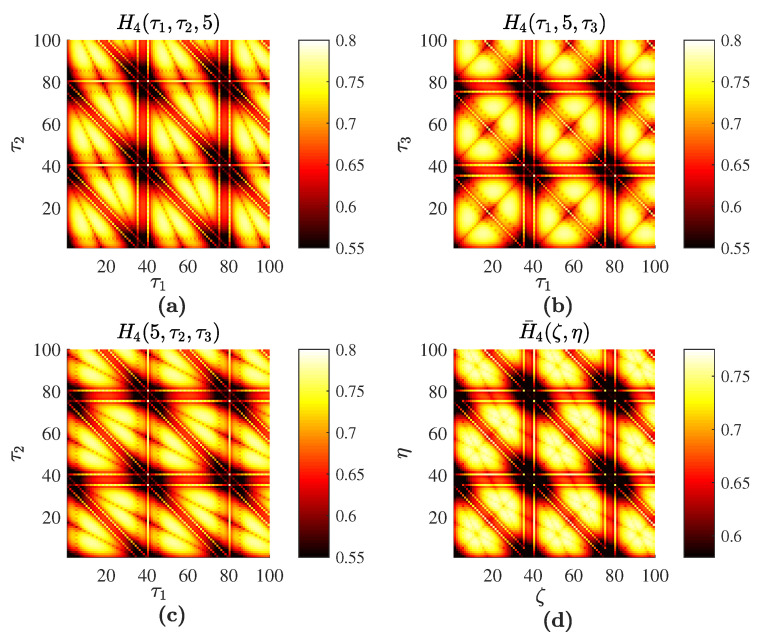
Permutation entropy for the sine wave: $H_{4}\left(\tau_{1},\tau_{2},5\right)$ is depicted in (**a**); $H_{4}\left(\tau_{1},5,\tau_{3}\right)$ in (**b**); $H_{4}\left(5,\tau_{2},\tau_{3}\right)$ in (**c**); and the averaged ${\bar{H}}_{4}\left(\zeta ,\eta \right)$ in (**d**); Numerical values of permutation entropy are indicated in color bars.

The sine wave used in this computational experiment is generated by the formula $x_{t}=\sin\left(\frac{2\pi t}{40}\right)$, t=1,…, 10,000; *L* is set to 100. The optimal embedding dimension is determined by FNN; D=4. Full sort yields the optimal set of time delays $\tau_{1}=\tau_{2}=\tau_{3}=5$. Planar projections $H_{4}\left(\tau_{1},\tau_{2},5\right)$, $H_{4}\left(\tau_{1},5,\tau_{3}\right)$, $H_{4}\left(5,\tau_{2},\tau_{3}\right)$ and the averaged ${\bar{H}}_{4}\left(\zeta ,\eta \right)$ are shown in Figure 1.

**Figure 2:**
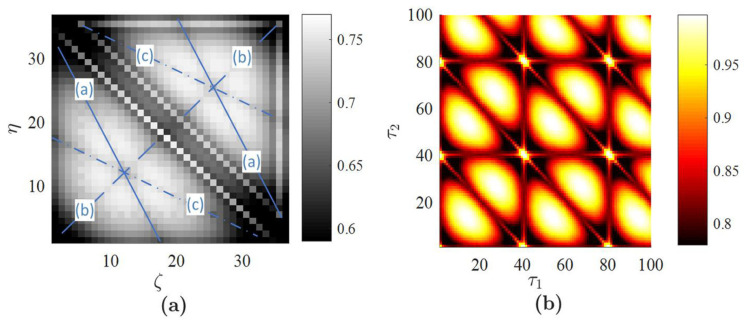
The geometric structure of a single periodic cell produced by the averaged PE reveals the three different inclination lines from three planar projections of the Permutation entropy (PE). (**a**) The PE reconstructed by the technique presented in [44] is depicted in (**b**).

It is well known that planar PE projections of the sine wave do exhibit a periodic pattern of periodic and symmetric cells (the size of these cells equals the ratio between the sampling frequency and the period of the sine wave) [44]. The averaged ${\bar{H}}_{4}$ based on the optimal embedding into the delay coordinate space also reveals the periodicity of the embedded time series. All three images $H_{4}\left(\tau_{1},\tau_{2},5\right)$, $H_{4}\left(\tau_{1},5,\tau_{3}\right)$ and $H_{4}\left(5,\tau_{2},\tau_{3}\right)$ are comprised of periodic cells with the same geometric boundaries. Though the structure of the geometric pattern in every cell is similar, the distortion of the image and the angles of the characteristic inclination lines are different (Figure 1a–c). Each cell in the averaged image yields traces of the three different inclination lines (Figure 1d). Thus, in principle, it is possible to read the optimal embedding dimension from the averaged image. The three inclination lines visible in each cell of Figure 1d suggest that the averaged image is comprised of three plain PE images, which in turn suggests that the optimal embedding dimension is four (Figure 2).

Computational experiments are continued with the sine wave using the technique presented in [44] (Figure 2b). The structure of the reconstructed image reveals the periodicity of the sine wave. However, the complexity of the image in Figure 2b is lower than the complexity of PE images depicted in Figure 1. This can be explained by the fact that the ordinal patterns are divided into 24 bins in Figure 1a–c versus six bins in Figure 2b.

### 4.2. The Rössler Time Series

**Figure 3:**
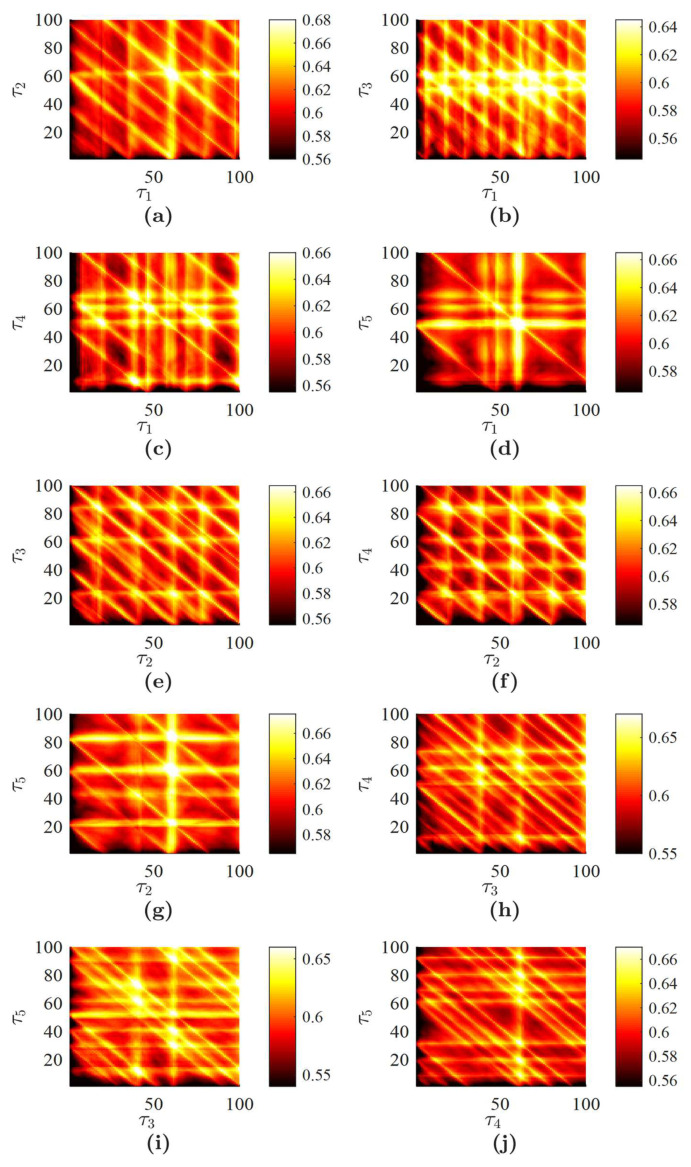
All possible planar projections of PE for the Rössler time series (D=6): (**a**) $H_{6}\left(\tau_{1},\tau_{2},42,21,23\right)$; (**b**) $H_{6}\left(\tau_{1},11,\tau_{3},21,23\right)$; (**c**) $H_{6}\left(\tau_{1},11,42,\tau_{4},23\right)$; (**d**) $H_{6}\left(\tau_{1},11,42,21,\tau_{5}\right)$; (**e**) $H_{6}\left(38,\tau_{2},\tau_{3},21,23\right)$; (**f**) $H_{6}\left(38,\tau_{2},42,\tau_{4},23\right)$; (**g**) $H_{6}\left(38,\tau_{2},42,21,\tau_{5}\right)$; (**h**) $H_{6}\left(38,11,\tau_{3},\tau_{4},23\right)$; (**i**) $H_{6}\left(38,11,\tau_{3},21,\tau_{5}\right)$; (**j**) $H_{6}\left(38,11,42,\tau_{4},\tau_{5}\right)$.

The Rössler system is a paradigmatic model of chaotic dynamics [45,46]:

(7) $$\left\{\begin{array}{c} \frac{dx}{dt}=-y-z,\\ \frac{dy}{dz}=x+ay,\\ \frac{dz}{dt}=b+z\left(x-c\right), \end{array}\right.$$

where constants a,b,c are set to a=0.1,b=0.1,c=14. The Rössler time series is generated by integrating the three coupled ordinary differential equations and by selecting every tenth value of *x* in the time domain 1≤t≤1000 (the time step is set to 0.01). FNN yields the optimal embedding dimension D=6; *L* is set to 100. Full sort yields the set of optimal time lags {38,11,42,21,23}. All planar projections of PE produced by fixing three of the five delays at the optimal values are illustrated in Figure 3.

**Figure 4:**
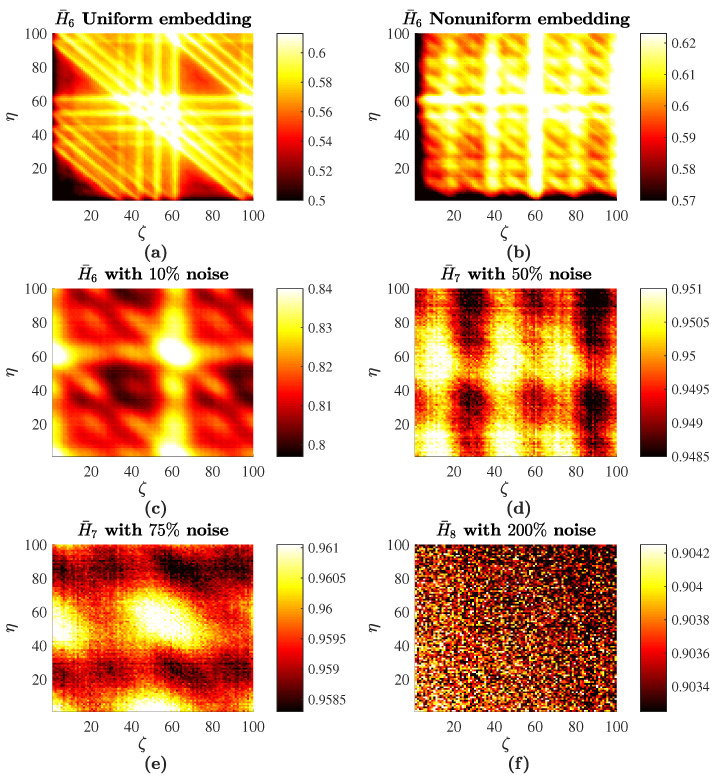
Averaged PE for the Rössler time series: (**a**) uniform embedding with no additive noise; (**b**) non-uniform embedding with no additive noise; (**c**) ${\bar{H}}_{6}$ with 10% noise; (**d**) ${\bar{H}}_{7}$ with 50% noise; (**e**) ${\bar{H}}_{7}$ with 75% noise; (**f**) ${\bar{H}}_{8}$ with 200% noise.

Finally, the averaged image of PE ${\bar{H}}_{6}\left(\zeta ,\eta \right)$ is depicted in Figure 4b. It can be observed that ${\bar{H}}_{6}$ integrates specific features represented in planar projections of PE and provides a single image representing the nonlinear properties of the Rössler time series. However, it is impossible to reconstruct the optimal embedding dimension from Figure 4a. First of all, this is due to the complexity of the time series. Secondly, the number of averaged images is large due to the fact that the optimal embedding dimension is large, as well. The Rössler time series represents a chaotic oscillator. Nevertheless, it is possible to assess an average period of chaotic oscillations (which is around 60 time steps in our computational experiment). The high-PE bands at delays of 60 (Figure 4b) denote this average period of oscillations (compare to Figure 1).

The averaged image ${\bar{H}}_{6}\left(\zeta ,\eta \right)$ can be compared with a similar reconstruction of PE with non-ideal parameters of the embedding. Uniform embedding yields the set of optimal time delays {9,9,9,9,9}; the optimal embedding dimension is the same (D=6; Table 2). The averaged ${\bar{H}}_{6}\left(\zeta ,\eta \right)$ produced by uniform embedding is depicted in Figure 4a. It is clear that the uniform embedding produces a much more regular image of averaged PE if compared to the non-uniform embedding. The high-PE bands at delays around 60 are still visible in images produced both by uniform and non-uniform embedding. However, the pattern of PE in Figure 4b is much less regular compared to the one in Figure 4a.

All real-world time series are contaminated by noise. Therefore, it is important to investigate the effects induced by noise on the proposed scheme of PE visualization. Computational experiments are continued with the Rössler time series, but different realizations of the Gaussian random noise with zero mean are added to it. The standard deviation of the random noise is set to $\frac{\beta R}{100\%}$, where β is the noise level in percent; *R* is the range of the stationary Rössler time series (the difference between the maximum and the minimum values of the time series in the observation window). ${\bar{H}}_{6}$ for the Rössler time series with different noise levels are depicted in Figure 4 (intermediate images of all possible plain projections of PE are omitted for brevity). The deterministic components of PE are gradually lost when the signal is completely buried in noise. ${\bar{H}}_{6}$ with 200% noise (Figure 4f) represents the image of random noise. It is known that PE is robust to noise [47]. However, the amount of noise added to the deterministic time series is so high that the time series becomes a random time series and correlations between data points are lost completely. It is interesting to observe that the average PE is more acutely impacted by noise when one of the coordinates (ζ,η) is low. Note that the addition of noise to the deterministic system induces the change of the optimal embedding dimension and optimal delays in the resulting time series. Therefore, the optimal embedding dimension and the set of optimal time delays are reconstructed for every different noise level (Table 2). Though PE is not a good measure to quantify the structural complexity, it is well suited to assess the randomness of a time series (PE increases with respect to noise level) [48]. This effect is clearly observed in Figure 4 when the range of PE raises from [0.58;0.63] up to [0.9937;0.9940].

The presented algorithm for plotting the averaged PE is based not only on the optimal embedding dimension, but also on the set of optimal time delays. Non-uniform embedding results in a different pattern if compared to uniform embedding (Figure 4a,b). However, it is also important to answer a question if the process of optimizing time delays yields any benefit aside from the averaging effect. In other words, it is important to compare PE patterns generated by the optimal non-uniform set of time delays with patterns generated by a set of delays generated by a random number generator.

**Figure 5:**
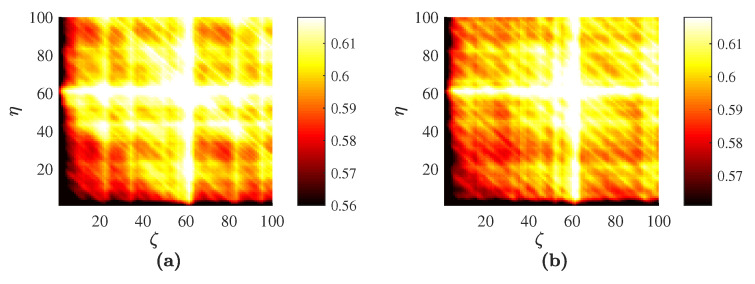
Averaged PE for the Rössler time series with no additive noise: (**a**) the pattern produced by a random set of time delays {7,18,39,27,12}; (**b**) by a random set of time delays {30,40,16,9,29}.

Computational experiments are continued with the Rössler time series without the additive noise. A standard random number generator is used to produce 10 real numbers uniformly distributed in the interval [0,1]. Every number is multiplied by 50 and rounded, resulting in two sets of random time delays: {7,18,39,27,12} and {30,40,16,9,29}. The averaged ${\bar{H}}_{6}$ for each set of random time delays are depicted in Figure 5.

It can be seen that particular values of time delays do have a strong impact on the averaged pattern of PE. The averaged PE inherits the properties of individual planar sections of PE. The geometric coordinates of the particular set of time delays in the multi-dimensional coordinate space define specific features of planar projections, which influence the averaged image. The high-PE bands at delays of 60 are still clearly expressed in Figure 5a,b. However, the averaged PE patterns are very much different; and the proposed technique for plotting the averaged PE is particularly based on the set of optimal time delays.

### 4.3. Real-World Time Series

**Figure 6:**
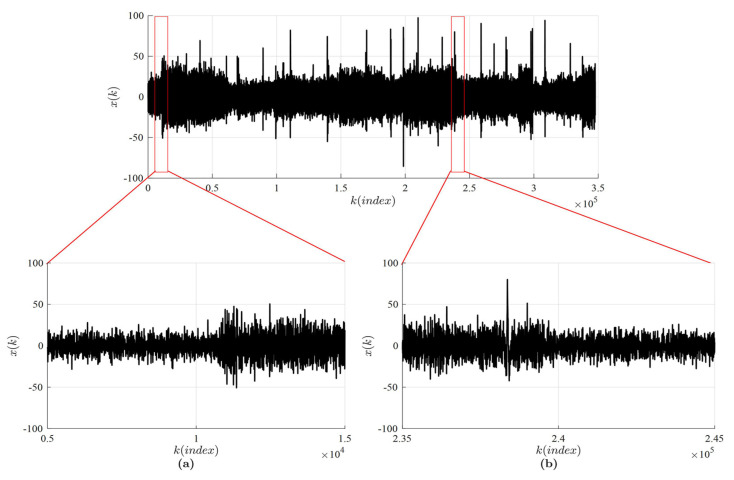
The Electroencephalogram (EEG) signal available from the Brain/Neural Computer Interaction (BNCI) Horizon 2020 project database [49]. Insets (**a**) and (**b**) are used to depict the zoomed parts of the signal.

Computational experiments are continued with the EEG signal available from the Brain/Neural Computer Interaction (BNCI) Horizon 2020 project [49] (A01 time series from dataset P300 Speller); the graphical representation of the signal is shown in Figure 6.

Two different intervals are selected from the same Electroencephalogram (EEG) signal: 1≤k≤100,000 (Interval A) and 200,001≤k≤300,000 (Interval B). Every second measurement point is skipped in both intervals in order to make the optimal time delays smaller (the number of remaining points in Intervals A and B is 50,000).

**Figure 7:**
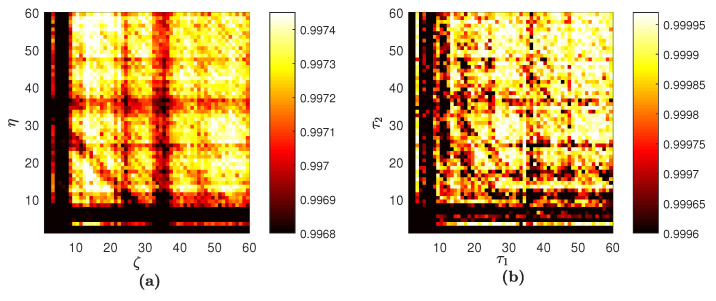
Digital images of PE reconstructed for Interval A. The proposed scheme yields the image in (**a**). The scheme without the assessment of the optimal embedding dimension and the optimal set of time lags results in the image in (**b**).

FNN yields the optimal embedding dimension D=5 for Interval A. The set of optimal time lags is $\left\{\tau_{1}=6,\tau_{2}=9,\tau_{3}=16,\tau_{4}=6\right\}$; the parameter *L* is set to 60. The averaged PE for Interval A ${\bar{H}}_{5}$ is depicted in Figure 7a.

Computational experiments are continued with Interval A, but now, PE is visualized using the technique proposed in [44]. The embedding dimension *D* is always three; time delays $\tau_{1}$ and $\tau_{2}$ are varied from 1–*L* (Figure 7b). Some important observations can be done by comparing the two digital images of PE in Figure 7. First of all, the process of averaging makes some features of the PE pattern appear cleaner. However, the averaging results in some information loss; for example, the stripe at $\tau_{2}=5$ in Figure 7b is not visible in Figure 7a. Clearly, some of the structure of the PE pattern in Figure 7a is replicated in Figure 7b, albeit with more noise. Furthermore, the difference in the PE values is a consequence of different *D*; and the fact that ordinal patterns are being divided into 120 bins in Figure 7a versus six bins in Figure 7b. The straightforward computation of PE (as shown in Figure 7b) yields a high level of noise what results in a high uncertainty of PE reconstruction. The improvement in the signal-to-noise of our PE reconstruction is due to the fact that the proposed algorithm averages over many two-dimensional images.

**Figure 8:**
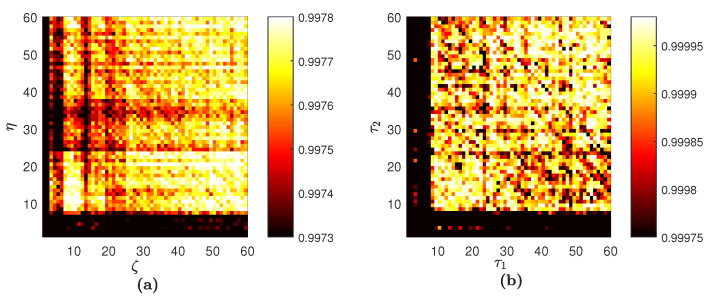
Digital images of PE reconstructed for Interval B. The proposed scheme yields the image in (**a**). The scheme without the assessment of the optimal embedding dimension and the optimal set of time lags results in the image in (**b**).

Analogous computations are performed with Interval B. FNN yields the optimal embedding dimension D=5 again, but the set of optimal time lags is now $\left\{\tau_{1}=5,\tau_{2}=6,\tau_{3}=14,\tau_{4}=6\right\}$. The averaged PE for Interval B ${\bar{H}}_{5}$ is shown in Figure 8a. The old visualization scheme for Interval B yields the digital image in Figure 8b. This confirms again that the optimal embedding dimension and the optimal set of time delays play a pivotal role in a representative visualization of PE. Comparison of the graphical features in digital images of PE represented in Figure 7a and Figure 8a helps to identify the differences occurring in the evolving time series. Such comparisons are much more difficult with the old visualization scheme (Figure 7b and Figure 8b).

## 5. Discussion

A novel visualization scheme for permutation entropy is presented in this paper. This scheme matches permutation entropy with the topological characteristics of the investigated time series: the optimal embedding dimension and the optimal set of time delays. The proposed scheme is based on non-uniform attractor embedding and uses different time delays, but results in a single digital image.

The proposed algorithm is based on the averaging of all possible plain projections of the permutation entropy measure in the multi-dimensional delay coordinate space. Such an approach is a natural extension of the technique used for the quantification of the phase space occupied by the reconstructed attractor [41]. The proposed scheme extends the visualization of permutation entropy from a three-dimensional phase space (with two time delays) to a multi-dimensional phase space.

The proposed scheme is well suited for real-world time series contaminated by the additive noise. Arithmetic averaging of plane projections reduces the optical effects induced by the additive noise and increases the clarity of specific geometric features (which can be used for the interpretation of the investigated time series). It is well known that permutation entropy can be used to identify couplings between time series [50]. The applicability of the proposed visualization scheme for the identification of couplings and synchronization between time series remains a definite objective of future research.

## Figures and Tables

**Table 1:** Schematic diagram for PE at D=5.

$\tau_{1}$	$\tau_{2}$	$\tau_{3}$	$\tau_{4}$	$H_{5}\left(\tau \right)$
1,…,L	1,…,L	$\tau_{3}^{★}$	$\tau_{4}^{★}$	$H_{5}\left(\tau_{1},\tau_{2},\tau_{3}^{★},\tau_{4}^{★}\right)$
1,…,L	$\tau_{2}^{★}$	1,…,L	$\tau_{4}^{★}$	$H_{5}\left(\tau_{1},\tau_{2}^{★},\tau_{3},\tau_{4}^{★}\right)$
1,…,L	$\tau_{2}^{★}$	$\tau_{3}^{★}$	1,…,L	$H_{5}\left(\tau_{1},\tau_{2}^{★},\tau_{3}^{★},\tau_{4}\right)$
$\tau_{1}^{★}$	1,…,L	1,…,L	$\tau_{4}^{★}$	$H_{5}\left(\tau_{1}^{★},\tau_{2},\tau_{3},\tau_{4}^{★}\right)$
$\tau_{1}^{★}$	1,…,L	$\tau_{3}^{★}$	1,…,L	$H_{5}\left(\tau_{1}^{★},\tau_{2},\tau_{3}^{★},\tau_{4}\right)$
$\tau_{1}^{★}$	$\tau_{2}^{★}$	1,…,L	1,…,L	$H_{5}\left(\tau_{1}^{★},\tau_{2}^{★},\tau_{3},\tau_{4}\right)$

**Table 2:** Optimal embedding dimensions and optimal time lags for the Rössler time series with different noise levels.

Noise Level	Dimension	The Set of Optimal Time Lags	Reference to Figure
0%	6	{9,9,9,9,9}	Figure 4a
0%	6	{38,11,42,21,23}	Figure 4b
10%	6	{37,45,17,38,6}	Figure 4c
50%	7	{36,16,14,28,35,36}	Figure 4d
75%	7	{33,15,33,25,25,16}	Figure 4e
200%	8	{49,41,15,36,33,16,40}	Figure 4f

## Abbreviations

The following abbreviations are used in this manuscript:

PE	Permutation Entropy
FNN	False Nearest Neighbors
EEG	Electroencephalogram
BNCI	Brain/Neural Computer Interaction
